# Interleukin-34–Induced Arg1^+^ Macrophages Play a Key Role in Breast Cancer Brain Metastasis

**DOI:** 10.1158/2767-9764.CRC-25-0639

**Published:** 2026-06-12

**Authors:** Xiaoqing Cheng, Khooshbu K. Patel, Brandon Zhou, Yiwei Fu, Ryan T. Cleary, Maureen Highkin, Jacob Hsia, Xiaohua Jin, Benjamin Kohn, Julie L. Prior, Megan S. Michie, Rui Sun, Xu Cheng, Liwei Bao, Jason Heth, Tusharika Rastogi, Amanda E.D. Van Swearingen, James D. Quirk, Ian S. Hagemann, Aki Morikawa, Nathan M. Merrill, Sofia Diana Merajver, Qingyun Li, Katherine Schwetye, Vaibhav Jain, Simon G. Gregory, Albert H. Kim, Ron Bose

**Affiliations:** 1 Division of Oncology, Department of Medicine, Washington University School of Medicine, St. Louis, Missouri.; 2 Duke Molecular Physiology Institute, Duke University School of Medicine, Durham, North Carolina.; 3 Department of Neurological Surgery, Washington University School of Medicine, St. Louis, Missouri.; 4 Mallinckrodt Institute of Radiology, Washington University School of Medicine, St. Louis, Missouri.; 5 Department of Internal Medicine, University of Michigan, Ann Arbor, Michigan.; 6 Department of Neurosurgery, University of Michigan, Ann Arbor, Michigan.; 7 Duke Center for Brain and Spine Metastasis, Duke University School of Medicine, Durham, North Carolina.; 8 Duke Cancer Institute, Duke University School of Medicine, Durham, North Carolina.; 9 Alvin J. Siteman Cancer Center, Washington University School of Medicine, St. Louis, Missouri.; 10 Department of Pathology and Immunology, Washington University School of Medicine, St. Louis, Missouri.; 11 Department of Neuroscience, Washington University School of Medicine, St. Louis, Missouri.; 12 Hope Center for Neurological Disorders, Washington University School of Medicine, St. Louis, Missouri.; 13 Department of Genetics, Washington University School of Medicine, St. Louis, Missouri.; 14 The Preston Robert Tisch Brain Tumor Center, Duke University School of Medicine, Durham, North Carolina.; 15 Department of Neurosurgery, Duke University School of Medicine, Durham, North Carolina.; 16 The Brain Tumor Center, Washington University School of Medicine, St. Louis, Missouri.

## Abstract

Brain metastases occur in up to 40% of patients with stage IV breast cancer, and the cerebellum is a common site for metastases in HER2-positive breast cancer. We developed a syngeneic, immunocompetent mouse model of breast cancer brain metastases (BCBM) by stereotactically injecting murine breast cancer organoids into the cerebellum. Spatial transcriptomics on these brain metastases revealed that breast cancer cells produce interleukin-34 (IL34), which induces ARG1^+^ macrophages at the invading edge of metastases. IL34 expression in human brain metastasis samples was measured in two independent datasets, and IL34 was expressed widely in both HER2^+^ and HER2^−^ human BCBM. Remarkably, IL34-deficient breast cancer cells failed to establish tumors in the mouse cerebellum. Furthermore, treatment with a blocking antibody against the IL34 receptor, CSF1R, led to tumor shrinkage, highlighting the translational potential of these findings, as a CSF1R-blocking antibody is FDA-approved for another indication, graft-versus-host disease.

**Significance:**

Targeting IL34–CSF1R is a potential new approach to treat BCBM and could be combined with existing therapies. IL34 expression is widely found in human BCBM.

## Introduction

Breast cancer brain metastases (BCBM) are an emergency and possibly catastrophic event for patients with breast cancer. Up to 40% of patients with stage IV breast cancer will develop BCBM (1, 2). Two aggressive subtypes of breast cancer, HER2^+^ and triple-negative breast cancer, are the most likely to cause BCBM, but they do so with different clinical patterns (1). Triple-negative BCBMs tend to be distributed throughout the brain (3), and they tend to occur when the patient also has cancer progression throughout the body, both inside and outside the central nervous system (CNS). In contrast, HER2^+^ BCBM has an increased predilection to metastasize to the cerebellum and will often occur when breast cancer outside of the CNS is under good control (3, 4). HER2^+^ BCBM can metastasize to other brain regions, but strikingly, in the German BCBM registry, 60% of the patients with HER2^+^ BCBM had metastases to the cerebellum (4). Cerebellar metastases pose a high risk to patients because of their proximity to the fourth ventricle and brainstem, which can cause obstructive hydrocephalus or affect breathing and other essential body functions. The differences in whether metastatic growth occurs outside the CNS are attributed to the effect of trastuzumab and other HER2-targeted monoclonal antibodies, which are highly effective outside the CNS but cannot readily penetrate the intact blood–brain barrier (BBB; ref. 5).

To model cerebellar BCBM from HER2^+^ breast cancer, we developed an immunocompetent, syngeneic transplantation strategy in which breast cancer organoids obtained from a C57BL/6J (B6) mouse are injected into another B6 mouse. Although FVB strain mice have traditionally been favored in breast cancer research, the differences in breast cancer development between B6 and FVB strains are small (6). Moreover, B6 mice are widely used for immunology research, and numerous transgenic and knockout strains are available in this strain’s background. Transplanting organoids between syngeneic mice provides an opportunity to separately genetically manipulate the “seed and soil” (7) of cerebellar BCBM. The “seed” is the breast cancer cell or organoid in this case, and the “soil” is the microenvironment of the cerebellar BCBM. Although liver metastasis has been studied using transplanted human colorectal cancer organoids (8), to our knowledge, this is the first study to transplant genetically engineered murine organoids to study BCBM.

The microenvironment of the cerebellum has unique features but also shares common features with other regions of the brain. Transcriptionally defined subpopulations of glial cells specific to the cerebellum have been described (9). Additionally, the neuronal cell types of the cerebellum are unique, with Purkinje neurons notable for their large dendritic arbors and small granule cell neurons, which are highly numerous. In fact, the cerebellum constitutes only 10% of the brain mass but contains more than 50% of the brain’s neurons, due to the very large number of these granule cell neurons (10). Common features include the presence of reactive glia and astrocytes in BCBM in the cerebellum and other brain regions (11). Furthermore, astrocytes stimulated with type I IFN can recruit monocytic myeloid cells, including macrophages, to the BCBM (12). Understanding this immune cell infiltration and its interactions with the microenvironment of the cerebellum when HER2^+^ BCBMs develop and grow is a major focus of this study.

A key mechanism driving macrophage infiltration is the interleukin-34 (IL34)–CSF1R signaling axis, which supports the recruitment, survival, and polarization of macrophages and microglia. IL34, produced by tumor and stromal cells, engages CSF1R on myeloid cells, fostering an immunosuppressive microenvironment that promotes tumor growth. These macrophage-rich infiltrates are a common feature across brain metastases and are thought to play a central role in shaping the tumor-permissive niche. Understanding how IL34–CSF1R signaling contributes to BCBM is another focus of this study.

In this study, we developed a model for HER2^+^ BCBM to the cerebellum in an immunocompetent mouse and performed spatial transcriptomics to characterize the brain tumor microenvironment (TME). We identified IL34 secretion by the breast cancer cells inducing ARG1^+^ macrophages to the periphery of the metastasis in which tumor invasion occurs. Because CSF1R is the receptor for IL34, we treated mice with established cerebellar BCBM with a CSF1R blocking antibody and observed shrinkage of the brain metastases. These findings have immediate translational potential because, in August 2024, the FDA approved a blocking CSF1R monoclonal antibody, axatilimab, for the treatment of another cancer-related condition, chronic graft-versus-host disease (13). Our findings suggest that targeting IL34–CSF1R–induced macrophages could potentially benefit patients with BCBMs.

## Materials and Methods

### Mice

The HER2^V777L^ transgenic mice were generated using TALEN-based genome editing as previously described (14, 15) and are deposited at The Jackson Laboratory (strain # 041201, RRID: IMSR_JAX:041201). The *PIK3CA*^H1047R^ mice in the C57BL/6J background are available at The Jackson Laboratory (strain # 041202, RRID: IMSR_JAX:041202). The *Trp53*^fl/fl^ mice were purchased from The Jackson Laboratory (strain # 008462, RRID: IMSR_JAX:008462). All animal experiments were approved by the Washington University Institutional Animal Care and Use Committee.

### Establishment of mammary gland organoids from murine breast cancers

Mammary gland tumors were resected from tumor-burdened *HER2*^V777L^; *PIK3CA*^*H1047R*^ (HP) or *HER2*^*V777L*^; *Trp53*^*fl/fl*^ (H53) mice. The tumors were minced, and tumor clusters were isolated by a modification of Ewald’s protocol (16). In brief, the minced breast tumors were enzymatically dissociated at 37°C in collagenase solution for organoids with agitation using a gentleMACS Octo Dissociator (RRID:SCR_020271), with heaters and the program, 37C_m_LDK_1, run twice. The dissociated tissues were centrifuged at 520 *g* for 10 minutes at 22°C. The supernatant was discarded, and the pellet was washed with advanced Dulbecco’s Modified Eagle Medium (DMEM)/F12, supplemented with 1× GlutaMAX, 1× HEPES, and 1× penicillin/streptomycin (+++ media) and centrifuged at 520 *g* for 10 minutes at 22°C. The cell pellet was treated with 1 mL of 1× RBC lysis buffer (Invitrogen eBioscience) for 5 minutes, followed by neutralization with 40 mL of PBS. After centrifugation, the cell pellet was treated with 4 mL of 4 U/mL of DNase I (Sigma-Aldrich, cat. #D4263) in +++ media, via shaking for 5 minutes. After DNase I treatment, 6 mL of +++ media was added, and the tube was centrifuged at 520 *g* for 5 minutes. The cell pellet was washed in 10 mL of +++ media, and after centrifugation, the pellet was resuspended in 90% Matrigel GFR (BD Biosciences, cat. #356231) and seeded in a six-well ultralow adhesion plate (Corning, cat. #3471). The embedded cells were overlaid with 3 mL/well mouse breast tumor medium (17): advanced DMEM/F12 (Gibco, cat. #12634010) supplemented with penicillin/streptomycin, 10 mmol/L of HEPES, GlutaMAX, 1× B27 (all from Thermo Fisher Scientific), 0.5× primocin (Invivogen), 125 μmol/L of N-acetyl-cysteine (Sigma, cat. #A9165-100G), 50 ng/mL of human epidermal growth factor (PeproTech or Thermo Fisher Scientific, cat. #AF-100-15-500UG), and 10% RSPO1-Noggin–conditioned medium. (The Noggin and R-spondin combined expression plasmid to generate conditioned media in the 293T cell line was a gift from Blair Madison, PhD, and Anil Rustgi, MD; ref. 18.) The mouse breast tumor medium was supplemented with the Rho kinase inhibitor, Y-27632 (AbMole, cat. #M1817), at 10 μmol/L for the first feeding after splitting. The growth medium was replaced every 3 to 4 days, and the organoids were passaged every 7 to 10 days by treatment with Cell Recovery Solution (Corning, cat. #354270), followed by digestion with TrypLE Express (Gibco TrypLE Express, cat. #12605028), mechanical dissociation, and re-embedding in fresh Matrigel and ultralow adhesion plates. Cell line authentication was not relevant as these are organoids prepared directly from primary tumors. The organoids were checked for contamination and tested negative for *Mycoplasma* contamination.

### Luciferase labeling

The organoid cells were transduced with a lentivirus-LUC luciferase reporter. Cloning selection was performed to isolate stable clones that expressed luciferase. Luciferase labeling was confirmed using quantification of *in vitro* bioluminescence signals.

### Stereotactic intracranial injections

The mice were anesthetized using 2% isoflurane with 100% oxygen for the duration of the operation. Prior to an incision above the base of the skull, the skin was sterilized with three alternating wipes of ethanol and betadine. The injections were done using a 28G needle with a standard 12-degree beveled needle point mounted on the mouse stereotactic frame, courtesy of the Albert Kim Lab. The coordinates we used for the cerebellum of the 5-month-old mice are AP = −6 mm from bregma, ML = −2.2 mm from midline, and DV = −1.5 mm from dura. After sterilization, the skull was exposed, and a small burr hole was drilled using a dental drill at the coordinates of the desired injection site according to Paxinos and Franklin’s mouse brain atlas, fifth edition. A microsyringe pump was used to deliver 3 μL of organoids, which had been dissociated into small clusters, into the cerebellum at a fixed rate of 0.4 μL/minute. Following injection, the needle was left in place for an additional 5 minutes to allow the injection solution to diffuse before slowly retracting. The injection site was sterilized with ethanol before the drilled hole was sealed with Gorilla Glue. The incision site was sutured, and the animals were allowed to recover on a heating pad before being returned to the animal cage for postoperative monitoring.

The organoids were removed from Matrigel using Cell Recovery Solution (Corning), washed, digested in TrypLE Express, and resuspended in DMEM. During the optimization process, different organoid cell numbers were injected into the cerebellum, ranging from 1,000 cells to 5,000 cells. The dissociated organoids for injection were prepared on the day of transplantation and kept on ice until the surgery was completed.

Power analysis: *n* = 5 or *n* = 10 mice were used for *in vivo* drug treatment experiments whenever possible, as prior power analysis showed that this provided >80% power for comparisons. Animals were randomized into drug treatment groups as follows: block randomization was performed based on the pretreatment size of the brain metastasis as determined via the MRI below.

### Small animal MRI

The mice underwent MRI starting 3 weeks after stereotactic injection. A gadolinium-based contrast agent was injected subcutaneously before imaging. While in the MRI scanner, the mice were anesthetized using isoflurane, and their heads were fixed in place using a bite bar and a custom 3D-printed nose cone. *In vivo* MRI studies were performed on a 9.4-T MR scanner (Bruker BioSpec) using an 86-mm diameter transmit coil and a four-channel mouse brain CryoProbe surface coil. The animals were anesthetized with a mixture of isoflurane in oxygen (4% for 90-second induction and 1% for maintenance), and the animal temperature was maintained at 37°C ± 1°C using a circulating water bath and heating pad placed under the animal. Respiration and temperature were monitored during MRI acquisition using SA Instruments (SA Instruments, Inc.) sensors. Immediately prior to imaging, each mouse was given a 300-μL subcutaneous injection of 2:10 Dotarem (Guerbet) diluted in saline. The MRI protocol consisted of a T2-weighted RARE image (TE = 35 milliseconds and TR = 2,750 milliseconds) and a T1-weighted RARE image (TE = 7.4 milliseconds and TR = 800 milliseconds). For each scan, 32 slices were acquired using a field of view of 16 × 16 mm^2^, an in-plane spatial resolution of 62.5 × 62.5 mm^2^, and a slice thickness of 500 mm. The total scanning time per animal was 20 minutes. Tumor size measurements were made by segmenting images using ITK Snap (http://www.itksnap.org/).

### Hematoxylin and eosin, IHC, and immunofluorescence staining

The tumor tissues were fixed with 10% formalin, embedded in paraffin, and cut into 5 μm sections. For hematoxylin and eosin, the slides were stained with hematoxylin, rinsed, and then counterstained with eosin before being mounted and imaged. For IHC and immunofluorescence (IF), the sections were deparaffinized and rehydrated with xylene substitute (Sigma-Aldrich A55597) and ethanol/PBS, followed by treatment with heat-activated antigen unmasking solution (Vector Laboratories ref. # H-3300). The slides were blocked in 5% BSA in PBS for 1 hour before incubation overnight with primary antibody (at 4°C). For IF staining, antibody binding was visualized with Alexa Fluor 488, 555, or 647 fluorochromes and then counterstained with DAPI-containing mounting media (Sigma). For IHC staining, the slides were treated with horseradish peroxidase (HRP)–conjugated antispecies secondary antibodies, followed by visualization with DAB substrate from Cell Signaling Technology and then counterstained with hematoxylin (Thermo Fisher Scientific). Immunostaining was performed using the following primary and secondary antibodies. The primary antibodies include human CD163 (Abcam, cat. #AB182422, RRID: AB_2753196), human ERBB2 (Cell Signaling Technology, cat. #2165S, RRID: AB_10692490), mouse ERBB2 (Cell Signaling Technology, cat. #2248, RRID: AB_2099242), ERα antibody (D-12; Santa Cruz Biotechnology, cat. #SC-8005, RRID: AB_627556), GATA-3 antibody (Santa Cruz Biotechnology, cat. #SC-268, RRID: AB_2108591), GFAP (Cell Signaling Technology, #80788), Iba1/AIF4T (E404W) antibody (Cell Signaling Technology, 17198S, RRID: AB_2820254), TMEM119 antibody (Cell Signaling Technology, 41134S, RRID: AB_3094467), arginase-1 antibody (Cell Signaling Technology, 93668S, RRID: AB_2800207), human IL34 (Abcam AB224734), murine IL34 antibody (R&D Systems, AF5195, RRID: AB_2124393), and human CD206 antibody (R&D Systems, cat. #AF2534, RRID: AB_2063019). The secondary antibodies for IF include anti-rabbit IgG (H+L) Alexa Fluor 555 (Invitrogen, cat. #A-31572, RRID: AB_162543), anti-goat IgG (H+L) Alexa Fluor Plus 488 (Invitrogen, cat. #A32814, RRID: AB_2762838), and goat anti–guinea pig (H+L) FITC (Fitzgerald Industries International, cat. #43R-1095, RRID: AB_10815085). The secondary antibodies for IHC include SignalStain Boost IHC Detection Reagent (HRP, Rabbit; Cell Signaling Technology, cat. #8114S, RRID: AB_10544930), SignalStain Boost IHC Detection Reagent (HRP, Mouse; Cell Signaling Technology, cat. #8125S, RRID: AB_10547893), and anti-sheep IgG (HRP-conjugated; R&D Systems, cat. #HAF016, RRID: AB_562591).

### *In vivo* CSF1R blocking antibody treatment

The CSF1R blocking antibody was purchased from BioXcell (Clone AFS98, cat. #BE0213, RRID: AB_2687699). For this experiment, the mice were stereotactically injected with 5,000 tumor organoid cells. After 17 days, treatment was started with the CSF1R blocking antibody injected intraperitoneally into the mouse every 2 days (19). The dosage on days 18, 20, 24, and 26 was 400 μg/mouse, whereas on days 22 and 28, it was 100 μg/mouse.

### *In vivo* BLI

For bioluminescence** i**maging (BLI) of live animals, the mice were injected intraperitoneally with 150 μg/g of D-luciferin (Gold Biotechnology, CAS # 115144-35-9, cat. #LUCK-100) in PBS, anesthetized with 2.5% isoflurane, and imaged with a charge-coupled device camera-based system (IVIS 50, PerkinElmer; Living Image version 4.3.1, exposure time 10–60 seconds, binning 8, field of view 12 cm, f/stop 1, open filter, ventral view). Luminescence was displayed as photons/sed/cm^2^/sr. The regions of interest (ROI) were defined manually over the lower abdomen using Living Image version 2.6, with measurements reported as photons/second.

### Near-infrared optical imaging

To prepare near-infrared (NIR)–trastuzumab, the fluorophore LS288 (4 eq) was activated using EDC/NHS ester chemistry and then conjugated to clinical-grade Herceptin (trastuzumab) via lysine labeling in a 0.1 mol/L NaHCO_3_ aqueous solution incubated at 4°C overnight. Zeba Spin Desalting Columns (0.5 mL, 40-K MWCO, Thermo Fisher Scientific) equilibrated with DPBS were used for the removal of excess free dye and purification of the NIR-trastuzumab (degree of labeling 2.2 or 1.5).

For *in vivo* fluorescence imaging, NIR-trastuzumab (1 mg/mL of antibody in 100 μL of DPBS) was injected into the tail vein of HP or H53 organoid-transplanted mice bearing tumors 24 hours prior to imaging. The NIR optical imaging of the live mice anesthetized with 2% isoflurane (vaporized in oxygen) was conducted using the Pearl Trilogy imager (800 nm channel, LI-COR) in the Washington University Molecular Imaging Center. LI-COR Image Studio software was used for measurement and image analysis.

### Visium spatial transcriptomics on mouse cerebellum brain metastasis

The tissue prepared for sectioning and staining starts with RNA quality assessment. RNA quality assessment of FFPE tissue blocks (RNA extraction and QC) needs to meet the DV200 quality score >30% to proceed with the assay using Agilent Bioanalyzer or TapeStation Instruments. The approved slide thickness is 5-μm sections compatible with the Visium CytAssist. The 20x resolution imaging on the ZEISS Axioscan 7 brightfield/fluorescence slide scanner was used for downstream Space Ranger Analysis. The sample/slide was prepared with de-cross-linking and library construction on a 6.5 mm × 6.5 mm capture area. The Visium CytAssist Spatial Gene Expression kit was used for FFPE (Mouse Transcriptome, 6.5 mm, 4 rxns, PN-1000521, 10x Genomics). The probe processes include probe hybridization/wash, probe ligation/wash, RNA digestion and tissue removal, probe extension and elution, preamplification, and cleanup and index PCR. CytAssist brightfield images were generated for downstream Space Ranger analysis. For sequencing information, 100 mol/L of total read pairs were targeted for each sample on the Illumina NovaSeq X Plus instrument (NovaSeq X Plus 10B at 300 cycles, 10 mol/L of targeted 2 × 150 clusters).

### Visium spatial data analysis

The initial Visium spatial data analysis was performed using 10x Genomics Space Ranger software (version 2.1.1). The Space Ranger includes two pipelines relevant to spatial gene expression experiments, which are mkfastq and count. Spaceranger mkfastq wraps Illumina’s bcl2fastq to demultiplex the sequencing runs and to convert barcode and read data into FASTQ files. Spaceranger count then takes a brightfield slide image and FASTQ files from mkfastq for alignment, tissue detection, fiducial detection, and barcode/unique molecular identifier (UMI) counting. After automated alignment and tissue identification, the spaceranger count pipeline utilized the full-resolution slide image to prepare data for visualization within Loupe Browser (10x Genomics: https://support.10xgenomics.com/single-cell-gene-expression/software/visualization/latest/what-is-loupe-cell-browser). The ROI of the tissue was identified and marked using Loupe Browser and subsequently selected for downstream analysis.

The output of the count pipeline contains an expression matrix, which was used for further analysis on the designated ROIs. For sample 1, the ROI consisted of 2,184 spots with a mean read depth of 30,825 reads per spot, whereas for sample 2, the ROI included 2,976 spots with a mean read depth of 28,271 reads per spot. The analyses were conducted using the Seurat (version 4.9.9.9086; source: vignettes/spatial_vignette.Rmd) package in R. The quality control and subsequent analyses were performed on the spot-level expression data along with the associated tissue slice image produced by Space Ranger. During the quality control step, the spots with the number of unique molecules less than 500 and the number of unique genes less than 10 were filtered out. After initial filtering, the final dataset retained 2,150 spots for sample 1, whereas the spot count for sample 2 remained unchanged. In Seurat, the filtered data are first normalized and scaled to account for the variance in sequencing depth across data points. On the normalized, scaled data, linear dimensionality reduction was performed by calculating 50 principal components using the most variably expressed genes in the spatial data. The spots were grouped into nine clusters for *de novo* cell type discovery using BayesSpace’s qTune and spatial cluster functions and graph-based clustering approaches, with visualization of spots being achieved through the use of clusterPlot (20).

The combined use of single-cell RNA sequencing (scRNA-seq) and spatial transcriptomics technologies on mouse brain specimens is expected to provide novel insights into the molecular, cellular, and spatial organization of distinct breast cancer metastases in relation to the surrounding TME. The publicly available single-cell datasets were used to identify the underlying composition of cell types (21–23). A reference-based deconvolution workflow—Spacex-R (24)—was applied to uncover cellular heterogeneity and the spatial arrangement of cell types in the samples, thus providing for each spot a probabilistic classification for each of the scRNA-seq–derived classes.

After spot deconvolution, differentially expressed genes (DEG) were identified using FindMarkers from the Seurat package (25) for regions such as the invasive region (near the tumor and near the normal area), tumor, and normal cerebellum. The top 10 upregulated and downregulated DEGs were visualized using a volcano plot (https://github.com/kevinblighe/EnhancedVolcano). Then, gene set enrichment analysis was performed with the identified DEGs using the fgsea R package (https://doi.org/10.1101/060012) utilizing the Molecular Signatures Database (26). MSigDb has 50 predefined hallmark gene signatures, which were leveraged to obtain the biological states or processes.

### IL34 CRISPR knockout organoid cell lines

IL34 knockout in breast cancer organoid cell lines was generated using the CRISPR–Cas9 system. Single-guide RNAs (sgRNA) targeting IL34 were designed and cloned into the lentiCRISPRv2 plasmid. The organoid cells were transduced with the sgRNA–Cas9 constructs via lentiviral delivery and selected with puromycin to enrich for successfully transduced populations. Knockout efficiency was confirmed at the protein level. IL34 secretion was further validated by ELISA, which showed a loss of IL34 expression in knockout organoid lines. Successfully edited organoids were expanded and maintained under standard culture conditions for subsequent functional assays.

*mIL34KO#1*: GTG​ATG​GAT​GTA​CTT​CTC​GA, *mIL34KO#2*: GAT​TGC​TGT​GCC​TTA​TGA​GG, *mIL34KO#3*: CAT​TGC​TGG​AGA​ACG​TAC​AG (sgRNAs).

### Human samples from Washington University School of Medicine

Patients with BCBM (*N* = 19) were retrospectively recruited from the CNS tumor bank at Washington University School of Medicine (WUSM) as part of a study that was approved by the Washington University Institutional Review Board (IRB # 2011-11001). The inclusion criteria were as follows: (i) histologically confirmed brain metastasis from breast cancer of any HER2, ER, and PR status, (ii) no prior radiotherapy, and (iii) female sex. Samples were collected by neurosurgical resection of brain metastasis performed between October 2018 and January 2025.

### RNA-seq analysis on patient-derived xenograft samples

The total RNA was extracted from patient-derived xenograft (PDX) samples. Libraries were prepared and sequenced on an Illumina sequencer model to generate paired-end reads. Raw reads were quality-checked, trimmed, and aligned to the human reference genome (GRCh38) using STAR, and gene counts were obtained with featureCounts. The counts were normalized using DESeq2’s variance-stabilizing transformation for downstream analyses, including principal component analysis and differential expression.

### Single-cell suspension preparation

The B6-HP tumor organoids were seeded the day prior to processing and dissociated with TrypLE the following day. Live and dead cells were assessed using Trypan blue staining. The cells were resuspended in PBS containing 0.04% BSA, with a viability of 82%. The sample was centrifuged at 400 × *g* for 5 minutes at 4°C and resuspended to a final concentration of 4,900 cells/μL in a total volume of approximately 90 μL.

### Single-cell library prep and sequencing

Utilizing the Chromium Next GEM Single Cell 3′ GEM, Library & Gel Bead kit version 3.3, and Chromium instrument, the cells were partitioned into nanoliter droplets to achieve single-cell resolution for a maximum of 10,000 to 15,000 individual cells per sample (10x Genomics). The resulting cDNA was tagged with a common 16-nt cell barcode and a 10-nt UMI during the reverse transcriptase reaction. Full-length cDNA from poly-A mRNA transcripts was enzymatically fragmented and size-selected to optimize the cDNA amplicon size for library construction (10x Genomics). The concentration of the 10x single-cell library was accurately determined through qPCR to produce cluster counts appropriate for the NovaSeq 6000 platform (Illumina). The sequence data were generated targeting 50,000 read pairs per cell, which provided digital gene expression profiles for each individual cell.

### Single-cell RNA sequence data analysis

The initial single-cell data analysis was performed using 10x Genomics software to convert barcode and sequencing read data into FASTQ files. Automated alignment, cell calling, Seurat analysis, and barcode/UMI counting were then performed using the FASTQ files. For secondary analysis of the mouse organoid scRNA-seq data, the Seurat package in R was used. The sample was filtered with quality control steps, including the removal of cells with low-quality UMI, genes, and mitochondrial contamination, followed by multiplate removal and ambient RNA exclusion. After quality control procedures were completed, we performed linear dimensional reduction, calculating principal components on the remaining cells using the most variably expressed genes in our dataset (27). Library size and/or the number of genes expressed across subsets of cells may necessitate the restriction of cells upon which the variably expressed genes were selected for inclusion when calculating principal components (28). The genes underlying the resulting principal components were examined in order to confirm that they were not enriched in genes involved in cell division or other standard cellular processes (28). Fifty significant principal components for downstream analyses were determined through methods mirroring those implemented by Macosko and colleagues (28), and these principal components were carried forward for two main purposes: to perform cell clustering and to enhance visualization (27). The cells were grouped into an optimal number of clusters for *de novo* cell type discovery using Seurat’s FindNeighbors() and FindClusters() functions and graph-based clustering approaches, with visualization of cells achieved through the use of the manifold learning technique Uniform Manifold Approximation and Projection for Dimension Reduction, which reduces the information captured in the selected significant principal components to two dimensions (arXiv 1802.03426; ref. 27). The dataset of Bach and colleagues (21) was used for cellular annotation for this sample.

## Results

### Organoid transplant model for HER2-positive BCBM to the cerebellum

**Figure 1.**
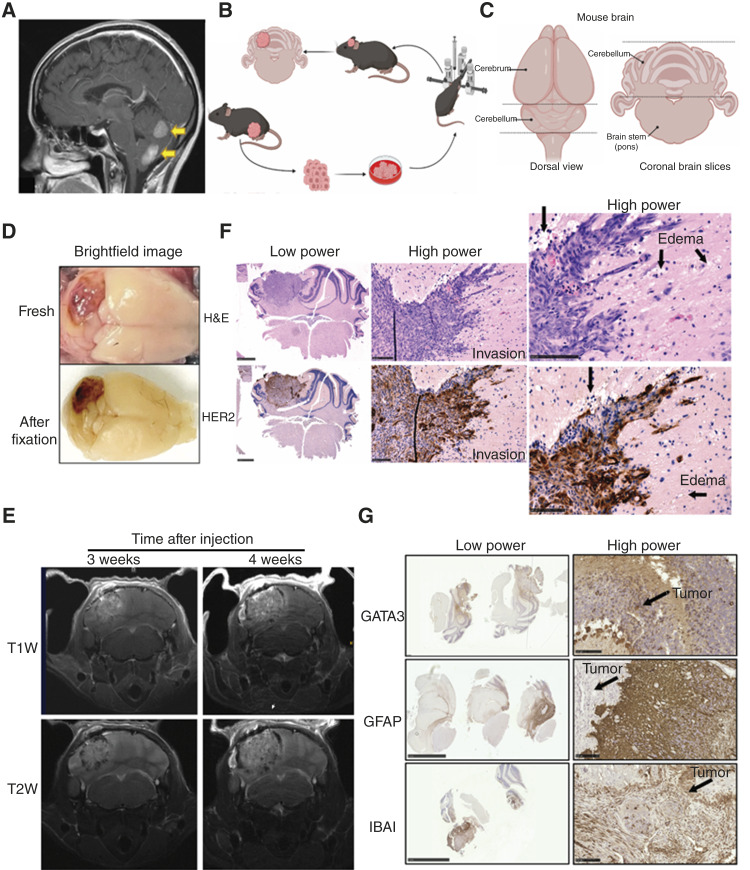
Organoid transplant model for HER2-positive BCBMs. **A,** MRI of a patient with HER2^+^ breast cancer shows two cerebellar metastases in the brain. **B,** Schema for establishing BCBM in mice. **C,** Dorsal and coronal brain slice views of the mouse cerebellum. **D,** Brightfield images of dissected and postfixation cerebellar metastasis. **E,** Contrast-enhanced T1-weighted and T2-weighted MRI of stereotaxic injection at 3 and 4 weeks after transplantation of HER2^+^ organoids in C57BL/6 syngeneic mice. **F,** Top, Hematoxylin and eosin (H&E) staining distinguishes the highly cellular breast cancer cells from the normal brain parenchyma, which is more eosinophilic and has sparse cell nuclei. Along the border of the lesion, areas of invasion and edema with microvacuolization can be seen. Bottom, IHC stained with ErbB2 antibodies confirms the overexpression of HER2 in the intracranial lesion, as well as HER2 expression in the invading cells. Scale bars of lower power images are 1 mm. Scale bars of high-power images are 100 μm. **G,** IHC staining of the BCBM with GATA3, GFAP, and IBA1 antibodies. Scale bars of lower power images are 5 mm. Scale bars of high-power images are 500 μm.

Figure 1A shows a patient with two large HER2^+^ BCBMs in the cerebellum. Remarkably, this patient did not have any metastases elsewhere in the brain. Her case and that of other similar patients motivated us to develop an animal model of cerebellar HER2^+^ BCBM to better understand, treat, and hopefully someday prevent this type of metastasis. An intracranial injection strategy (Fig. 1B) was chosen because of its feasibility and anatomic precision. Intracranial injection is widely used in studies of glioblastoma or BCBMs that use human PDXs (29). Using stereotactic injection equipment, the location and brain region of the BCBM can be precisely controlled. Furthermore, this direct injection approach yields very predictable timing and a high success engraftment rate for metastasis development. Although intracardiac injection models have been favored for studying BCBM (11, 30–32), it is more challenging to study cerebellar BCBM for several reasons. The blood supply to the cerebellum predominantly comes from the vertebral artery and the posterior circulation of the brain (10). Intracardiac injection delivers most of the breast cancer cells to the cerebrum and not to the cerebellum. Similarly, carotid artery injection primarily does not reach the posterior circulation of the brain. Developing a breast cancer cell line that homed specifically to the cerebellum would be desirable but is not currently available (33). The ideal model would be a transgenic mouse that spontaneously develops cerebellar BCBM; unfortunately, no such mouse model currently exists, to our knowledge.

We previously developed a transgenic mouse that overexpresses the human HER2 gene with a V777L activating mutation (14, 15). The use of HER2 transgenes with activating mutations is very common in the HER2 field. The first HER2 transgenic mouse carries the rat HER2 V664E activating mutation driven by the MMTV promoter (34). More recently, a Tet-inducible HER2 transgenic mouse also used this same rat HER2 V664E activating mutation (35).

We chose the human HER2 gene for our transgenic construct because trastuzumab is species-specific, recognizing human but not rodent HER2 (14, 15). Furthermore, our human HER2^V777L^ transgene is driven by an actin promoter, generating overexpression with intense circumferential membrane staining that is clinically scored as 3+ IHC staining (14, 36).

Our transgenic mouse has three additional important properties. First, it contains an Lox–STOP–Lox element generating conditional expression (15). Second, we found that the addition of a second genetic alteration, either a *PIK3CA*^H1047R^ activating mutation or a *TP53* deletion, dramatically accelerates tumorigenesis, matching genotypes seen in human patients with cancer (14). Third, we found that breast cancer organoids derived from these mice can be reimplanted into the mammary fat pad of syngeneic B6 mice and will form mammary tumors that spontaneously metastasize to the lungs of the recipient mice (14). Therefore, we hypothesized that the injection of these organoids into other sites in syngeneic mice could create models of other types of breast cancer metastases.

Figure 1B shows the schema used to generate cerebellar BCBM in mice. We used B6 mice expressing HP activating mutations or HER2^V777L^; *TP53*^*flox/flox*^ (H53). In brief, breast cancer primary tumors were established in either HP or H53 mice by injections of Cre-expressing adenovirus into the mammary gland; these tumors were harvested, and murine breast cancer organoid cultures were established from these tumors. These murine cancer organoids were luciferase-labeled and expanded *in vitro*. For the intracranial injections, HP or H53 organoids were dissociated into small clusters and stereotactically injected unilaterally into the cerebellum (Fig. 1B). In all, 1,000 or 5,000 cells were injected per mouse. Figure 1C shows the location of the cerebellum in the dorsal view of the intact mouse brain and in coronal brain slices or images. BCBM growth was confirmed using MRI at 3 weeks, and the mice were then sacrificed. After dissection and fixation, the cerebellar tumor had a hemorrhagic appearance distinct from the normal brain tissue (Fig. 1D).

We used MRI to monitor the metastatic tumor *in vivo* (Fig. 1E). Axial T1-weighted postcontrast MRI revealed interval growth of a hyperintense enhancing cerebellar lesion with irregular borders at the site of injection over 4 weeks (Fig. 1E). At 3 weeks after injection, the lesion was located above the fourth ventricle and brainstem. Although gradual growth was seen from 3 weeks after injection, the tumor mass was significantly larger at 4 weeks after injection, exerting pressure on the fourth ventricle (Fig. 1E).

Following histologic sectioning and staining, we identified several areas of interest around the lesion (Fig. 1F). Areas of invasion could be identified at the border of the BCBM. Tumor cells were depolarized and had a flattened cell morphology, resembling a motile phenotype [Fig. 1F (middle)]. In addition, areas of edema and microvacuolization were present at the edge of the metastasis [Fig. 1F (black arrows)], indicative of cancer-associated inflammation bordering the metastasis. To validate that the primary tumor subtype had been retained, the tumor was stained with HER2 antibody, and HER2 overexpression was confirmed [Fig. 1F (bottom)]. Most importantly, cells in areas of invasion retained their HER2 expression. Overall, this animal model provides a feasible and robust approach to study cerebellar BCBM, and we characterized it further.

### IHC characterization of HER2^+^ cerebellar BCBM

**Figure 2.**
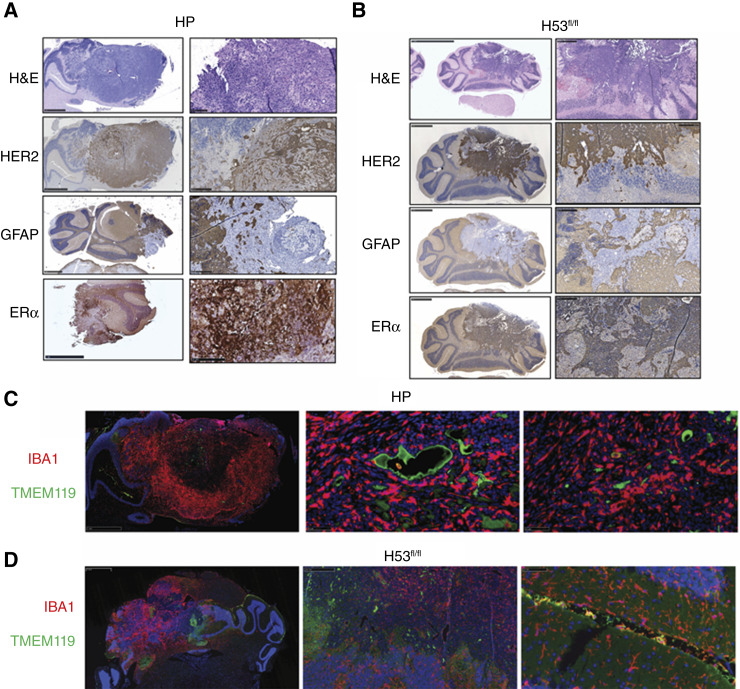
Characterization of cerebellum metastasis mouse model. **A,** Hematoxylin and eosin (H&E) and IHC stained with GFAP, HER2, and ERα antibodies in the cerebellum metastasis model of HP tumor organoid cells. Scale bars of low-power images are 5 mm. Scale bars of high-power images are 500 μm. **B,** H&E and IHC stained with GFAP, ERα, and HER2 antibodies in the cerebellum metastasis model of H53 tumor organoid cells. Scale bars of low-power images are 5 mm. Scale bars of high-power images are 500 μm. **C,** IF stained with IBA1 and TMEM119 antibodies in the cerebellum metastasis model of HP organoid cells. Scale bars of low-power images are 5 mm. Scale bars of high-power images are 500 μm. **D,** IF stained with IBA1 and TMEM119 antibodies in the cerebellum metastasis model of H53 tumor organoid cells. Scale bars of low-power images are 5 mm. Scale bars of high-power images are 500 μm.

We performed IHC staining on these BCBM for several markers. GATA3 is a transcription factor expressed by breast cancer cells and is present in the BCBM (Fig. 1G). GFAP is a marker of astrocytes and other glial cell types, including ependymal cells. GFAP expression is absent in these BCBMs (Figs. 1G, 2A, and B). Ionized calcium–binding adaptor molecule 1 (IBA1), also called allograft inflammatory factor 1, is a well-established marker for microglia/macrophages. The IBA1-positive cells were seen within the BCBM (Fig. 1G). The HP and H53 organoids are estrogen receptor α (ERα) positive (Supplementary Fig. S1A; ref. 14), and ERα expression is seen in these BCBMs (Fig. 2A and B). About half of the human HER2^+^ BCBMs are ER^+^ (2), and therefore, these HER2^+^ ER^+^ BCBMs recapitulate what occurs in patients. To further define the cell types present in these organoids, we performed scRNA-seq on the HP organoids (Supplementary Fig. S1B). We found that 89% of the cells in the HP organoids are breast cancer cells, 0.26% are fibroblasts, and 9.6% are other stromal cells, including endothelial cells and adipocytes. Lymphocytes and myeloid cells are <0.1% of the cells in the organoid preparation.

To further characterize the IBA1^+^ cells in these BCBM, we performed dual IF for both IBA1 and the microglia marker TMEM119 (Fig. 2C and D). We observed very little colocalization of IBA1 and TMEM119, suggesting that the IBA1^+^ cells may be bone marrow–derived macrophages (BMDM) and not microglia. However, we acknowledge that a recent study reported that TMEM119 expression is downregulated in a subset of brain metastasis–associated microglia (37); therefore, further evidence, such as transcriptomics data, is needed to determine the identity of the IBA1^+^ cells. We confirmed that the murine organoid cells injected to form these BCBMs did not contain IBA1^+^ cells (Supplementary Fig. S1). For the remainder of our experiments, we chose to focus on BCBM generated with the murine HP organoids.

### NIR-trastuzumab optical imaging of brain metastases

**Figure 3.**
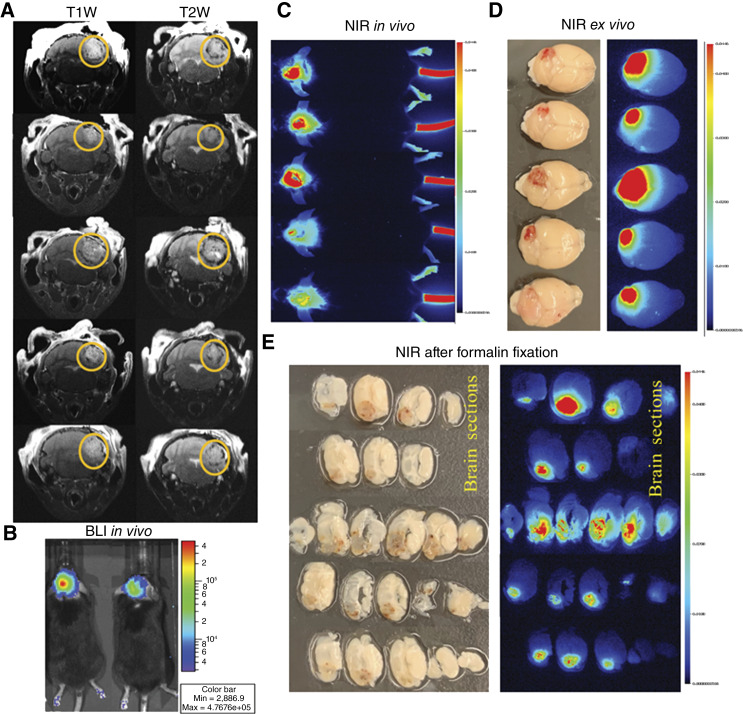
Herceptin optical imaging of brain metastases. **A,** Contrast-enhanced T1- and T2-weighted MRIs of stereotaxic injection at 3 weeks after transplantation of HP organoids in C57BL/6 syngeneic mice. **B,** Representative BLI images of the cerebellum tumor at 3 weeks after organoid cell transplantation of HER2^+^ tumor organoids. **C,** Live animal *in vivo* imaging of NIR fluorophore–labeled trastuzumab (NIR-trastuzumab). Mice were injected with 100 μg of NIR-trastuzumab via the tail vein 24 hours prior to this imaging. **D,** Left, Brightfield images of dissected cerebellum tumor at 3 weeks after transplantation. Right, Imaging of NIR-trastuzumab after dissection. **E,** NIR-trastuzumab imaging was performed after 24 hours of formalin fixation of mouse brain tissues. Left, Brightfield images of formalin-fixed cerebellum tumor slices. Right, Imaging of NIR-trastuzumab after formalin fixation in the same cerebellum tumor slices.

The BBB normally limits the penetration of many drugs and antibodies into the CNS. However, the BBB around brain metastases is abnormal and leaky and has been termed the blood–tumor barrier (5). We had previously imaged lung metastases and mammary tumors in HP mice with optical imaging using an NIR fluorophore conjugated to trastuzumab (14). To determine whether NIR-trastuzumab imaging was effective for these cerebellar BCBMs, we injected NIR-labeled trastuzumab into the tail vein of five cerebellar BCBM-bearing mice. MRI images in Fig. 3A confirm the presence of the cerebellar BCBM, and bioluminescence imaging (Fig. 3B) also demonstrated the presence of cerebellar BCBM. Twenty-four hours after NIR-trastuzumab injection, the mice were shaved and underwent *in vivo* imaging (Fig. 3C). The NIR signal was readily detected through the intact skin and skull of all five mice. NIR-trastuzumab has an advantage over bioluminescence imaging as it can be performed *ex vivo* on dissected or even on formalin-fixed tissues (Fig. 3D and E). This *ex vivo* signal is present because, unlike bioluminescence imaging, NIR imaging does not require ATP or living cells (38). Therefore, NIR imaging can be used to facilitate brain dissection. However, the NIR signal is lost when the tissue is exposed to organic solvents used in paraffin embedding and routine histology (39).

Overall, NIR-trastuzumab tumor-specific imaging is a potent tool for studying HER2^+^ BCBM. It demonstrates that monoclonal antibodies and conjugated antibodies can penetrate into these cerebellar BCBMs. Furthermore, labeling trastuzumab with other imaging agents, such as PET isotopes like ^89^Zr or ^64^Cu, can provide a wide range of options for imaging HER2^+^ BCBMs (39).

### Spatial transcriptomics reveals the role of Arg1 in the invasion clusters

**Figure 4.**
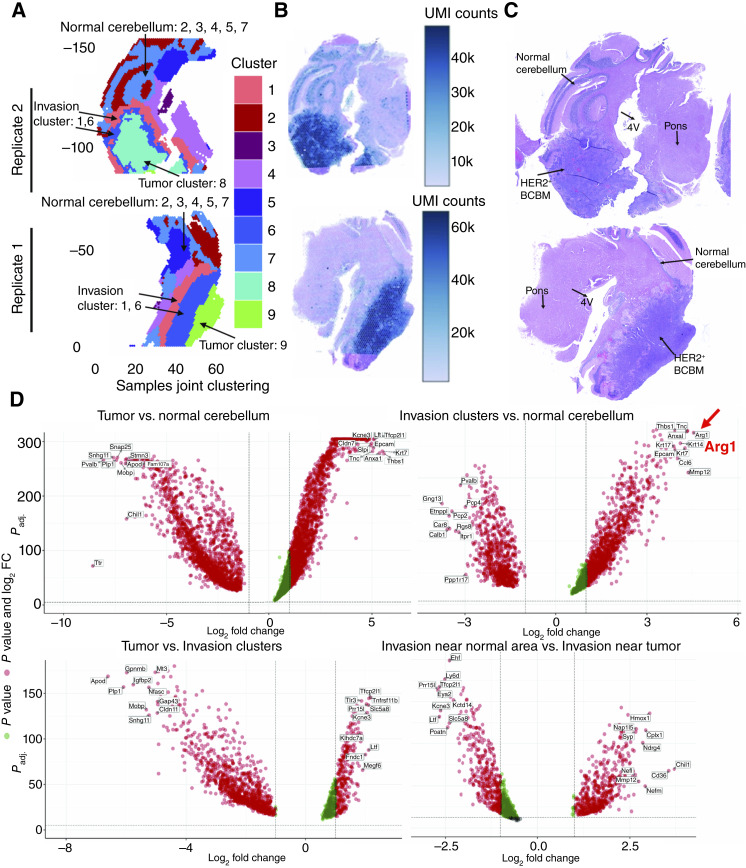
Spatial transcriptomics reveals the clusters of cells and genes in HER2^+^ BCBM. **A,** Sample joint clustering in the duplicate samples of spatial transcriptomics in the HER2^+^ breast cancer cerebellum metastasis model. The average expression of the whole genome in each cluster is provided in Supplementary Table S1. **B,** Tissue plot with spots colored by UMI count in duplicate samples. **C,** Hematoxylin and eosin staining of biological duplicate samples for spatial genome-wide sequencing. **D,** Volcano plot illustrating genes meeting cutoffs for differential expression [log-fold change (logFC2) >1, *P*_adj._ < 0.05] between tumor vs. normal cerebellum, tumor vs. invasion clusters, invasion clusters vs. normal cerebellum, and invasion clusters near the normal area vs. invasion near the tumor. A list of the significantly altered genes in replicate two is provided in Supplementary Table S2.

The TME plays a crucial role in the development, progression, and response to therapy in HER2^+^ breast cancer. The TME of HER2^+^ breast tumors is normally characterized by a complex interplay of various cell types, including cancer cells, stromal cells, immune cells, endothelial cells, and extracellular matrix components. HER2^+^ breast tumors often exhibit increased immune infiltration, with higher tumor-infiltrating lymphocyte levels than HER2-negative tumors (40). Therefore, we investigated how the HER2^+^ BCBM tumor interacts with the TME of the cerebellum. We generated spatial transcriptomics data from two biological replicates of the cerebellar BCBM generated from HP organoids. After QC prefiltration, qTune/qPlot, batch effect, and harmonizing the samples and clusters, we classified the samples into nine clusters (Fig. 4A; Supplementary Table S1). The nine cell clusters were further categorized as normal cerebellum, tumor cluster, and invasion cluster. In particular, the invasion cluster was differentiated into cluster 1, which faced the normal cerebellum, and cluster 6, which bordered the core of the tumor lesion, to help us better understand the progression of tumor invasion (Fig. 4A). The UMI count distinguished between the HER2^+^ BCBM tumor and the normal area (Fig. 4B), corresponding with the histologic morphology (Fig. 4C). The volcano plot highlighted the genes that were differentially expressed among tumor, invasion, and normal cerebellum clusters (Fig. 4D; Supplementary Table S2). Specifically, we set a comparison group between cluster 1, the invasive region adjacent to the normal cerebellum, and cluster 6, the region including both the invasive perimeter and the tumor lesion core, to differentiate the gradual occurrence of metastasis (Fig. 4D). Compared with the normal cerebellum and invasive clusters, many epithelial markers and inflammatory-associated genes were upregulated in the tumor clusters, including *Mmp3*, *Epcam*, *Krt7*, *Igha*, *Ighg2b*, *Igkc*, and *Anax1* (Fig. 4D). In addition, some monocarboxylate transporters like *Slc5a8* and cell–cell adhesion markers like *Col14a1*, *Fbln7*, and *Lum* were highly expressed in tumor clusters (Fig. 4D). Interestingly, *Ltf*, which is a protein product found in the secondary granules of neutrophils, was upregulated in tumor populations. Some brain-dominant expressed genes like *Clip1* and *Clip2* were also found in metastatic tumors, highlighting the unique molecular heterogeneity of the tumor cluster profile (Fig. 4D). The invasion clusters exhibited enhanced gene signatures associated with cell–cell adhesion (*Col5a1*, *Col6a3*, and *Col8a1*), angiogenesis (*Hmox1*), phagocytosis (*Arg1*), and metastasis (*Mmp12* and *Mmp13*; Fig. 4D). Importantly, *Arg1* gene expression was consistently upregulated in the invasion clusters across the replicate samples. *Arg1* is a marker of immunosuppressive macrophages, which can inhibit T-cell activation (41).

Consistently, several inflammatory genes are increased in the BCBM tumors, including *Cgas*, *Jak2*, *Stat1*, *Stat3*, and *Nfkb1* (Supplementary Fig. S2). High expression of *Trem2*, *TGFβ*, and *Ifngr1* in Supplementary Fig. S2D suggests that HER2^+^ BCBMs attract immunosuppressive cells like regulatory T cells, myeloid-derived suppressor cells, and tumor-associated macrophages, thereby creating an immunosuppressive TME. HER2 signaling can upregulate cytokines such as IL6 and chemokines like CCL2, promoting a proinflammatory and immunosuppressive environment conducive to tumor growth and metastasis. In fact, inflammation is the key step that will result in neurodegenerative disease (42) and primary brain tumors (43). Cerebellar granule cells undergo major processes of neural development and synaptic connections, which are sensitive to the chemokines and cytokines secreted by immunogenic or tumor cells. *Vegfa*, *Hif1a*, and *Hmox1* are highly expressed in the invasion clusters, correlating with increased angiogenesis (Supplementary Fig. S2E).

These results demonstrate that distinct gene expression panels exist in the tumor, normal cerebellum, and invasion clusters. *Arg1* in the invasion clusters suggests its role in prometastatic potency. However, it is unknown which cell types mediate the invasion by interacting with normal cells in the cerebellum.

### Distinguished enrichment of pathways and cell types in the spatial landscape

**Figure 5.**
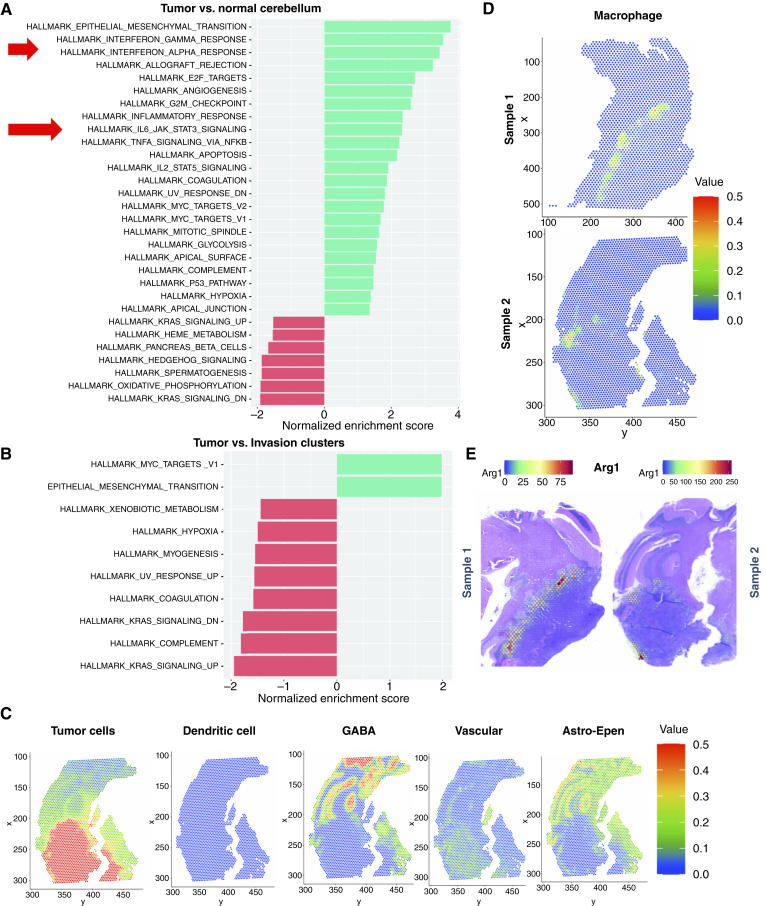
Enrichment of pathways and cell types in the spatial landscape of tumor, invading, and normal cerebellum clusters. **A,** Gene sets significantly enriched in tumors compared with normal cerebellum as identified via GSEA (*P* < 0.05). ES (enrichment score) and −log_10_ (*P* values) of pathways are shown. GSEA was performed using the hallmark gene sets in the Molecular Signatures Database (version 7.5.1). A list of the hallmark genes in replicate one is provided in Supplementary Table S3. **B,** Gene sets significantly enriched in tumors compared with invasion clusters as identified via GSEA (*P* < 0.05). ES (enrichment score) and −log_10_ (*P* values) of pathways are shown from GSEA performed using hallmark gene sets in the Molecular Signatures Database (version 7.5.1). A list of the hallmark genes in replicate one is provided in Supplementary Table S3. **C,** Integrated spatial distribution panel of cell types of prediction in duplicate samples. Cell types are identified by a combination of snRNA and spatial transcriptomics technology. **D,** Spatial distribution of macrophages as indicated in duplicate samples. **E,** Spatial distribution of Arg1 as indicated in duplicate samples.

HER2^+^ breast tumors can attract immune cells to the TME through various mechanisms, including the secretion of chemokines and cytokines. Tumor-infiltrating immune cells, such as T, B, natural killer, and dendritic cells, interact with HER2^+^ cancer cells and influence tumor growth, invasion, and metastasis (44). We tested whether HER2 activation in cancer cells can produce cytokines, chemokines, and growth factors that modulate the TME in the scenario of cerebellar metastasis. The GSEA analysis using the hallmark gene set indicated that tumor versus normal cerebellum DEGs were significantly enriched for genes related to inflammation, epithelial–mesenchymal transition (EMT), cell cycle, and metabolism (Fig. 5A and B; Supplementary Table S3). These data confirmed the distinct cluster properties in the tumor, normal cerebellum, and invasion edge.

The TME plays a critical role in shaping behavior and response to therapy in HER2^+^ BCBMs. Elucidating the complex interactions between HER2 signaling and the TME components is essential for developing novel therapeutic approaches to overcome treatment resistance and improve outcomes for patients with HER2^+^ BCBMs. We next differentiated the cell types by integrating our spatial transcriptomics data with scRNA-seq data (Fig. 5C). The tumor cell signature can be clearly seen in red in Fig. 5C (left). The γ-aminobutyric acid (neurons) and Astro-Epen–positive (glial) cell populations are mainly located in the normal cerebellum area. There are immune cells, like myeloid leukocytes and stroma cell types, enriched in the tumor cohort; however, we did not see a significant difference in the infiltration of T, B, and dendritic cells. Interestingly, macrophage cells are significantly upregulated in the invasion clusters (Fig. 5D), consistent with the *Arg1* spatial distribution (Fig. 5E).

In summary, we identified macrophages at the invading edge, overlapping with increased *Arg1* transcript expression seen in the spatial transcriptomics data. Understanding the interactions between HER2^+^ BCBM and the TME in the cerebellum is essential for developing effective therapeutic strategies for brain metastasis.

### Spatial transcriptomics panels reveal a cluster-type “gradient” as HER2^+^ tumor invades the cerebellum

**Figure 6.**
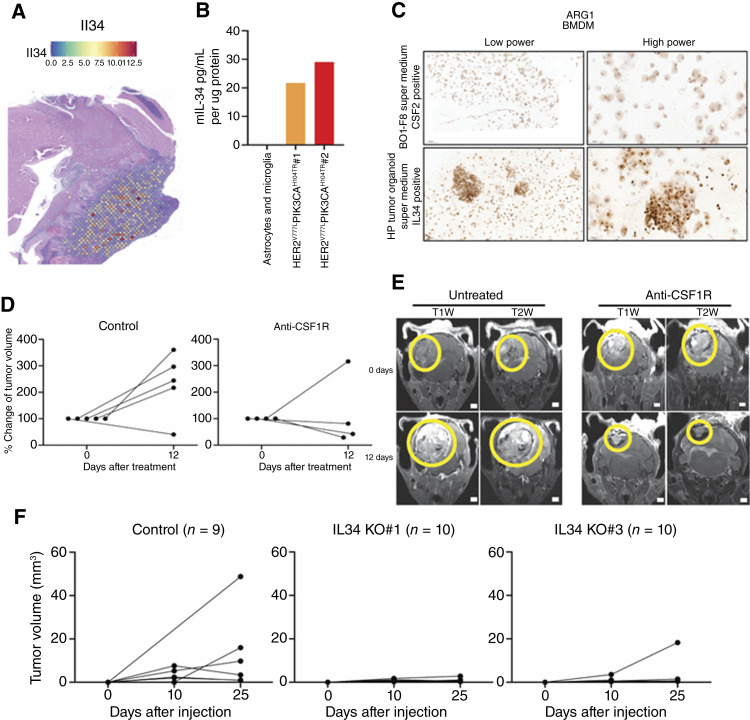
IL34-induced Arg1 expression at the invading edge in cerebellum metastasis of HER2^+^ breast cancer. **A,** Spatial feature plot of *IL34* in a representative murine tissue sample. **B,** ELISA test result of IL34 protein level from astrocytes and microglial cells, HP breast cancer organoid cells. **C,** Mouse BMDM was cultured with conditioned media supernatant from BO1 cells or HP tumor organoid cells for 24 hours, and the IHC staining of ARG1 was performed on cytospin slides of the cocultured cells. **D,** Percent change of tumor volume in the control and anti-CSF1R mAb mouse groups. **E,** The representative contrast-enhanced T1W and T2W images of MRI showed the cerebellar tumor volume change at 0 and 12 days after treatment in the control and anti-CSF1R mAb groups. Briefly, 5,000 cells from HP breast cancer organoids were injected into the mouse cerebellum as described in the methods. **F,** Change of tumor volume in the control and IL34 knockout mouse groups.

Previous studies have shown that ErbB2 and ErbB4 are constitutively expressed in granule cells during the first 2 to 3 weeks postnatally (45), suggesting that dimerization, such as ErbB2/ErbB4 or ErbB4/ErbB4, may be important for processes such as granule cell migration, neuronal connection, and synaptic formation. We analyzed gene expressions in specific cell subtypes. *HER2*, *EGFR*, and *HER3* were upregulated in the tumor clusters, along with increased downstream signaling genes, *Akt* and *Mapk1*, suggesting that a combination of HER2 with EGFR or HER3 was required to form functional receptors in cerebellar metastatic tumors (Fig. 6A). In contrast, *HER4* is mainly expressed in the normal cerebellum clusters, revealing its role in the normal cerebellum (Supplementary Fig. S2A). The EGF family ligands, *Egf*, *Nrg1*, and *Nrg2*, are expressed in both the normal cerebellum and the BCBM tumor (Supplementary Fig. S2B).

Particularly, *Tgfb1* expression levels are much higher than those of other cytokines (Supplementary Fig. S2D). In addition, *Ifngr*, a receptor of the type II class of IFNs, which are Jak-Stat-Gas pathway–specific cytokines, is mainly localized in the tumor area (Supplementary Fig. S2D). Both cytokines and growth factors can trigger Jak–Stat–Gas signaling. The STING pathway detects extracellular DNA, which can indicate either a foreign invader, such as a virus, or damage to host tissue or cells. HER2 recruits AKT1 to disrupt STING signaling and suppress antiviral defense and antitumor immunity (46).

EMT is the mechanism for invasion in many types of cancer. *Snail*, *Snail2*, *Twist1*, and *Cdh2* are increased in both tumor and invasion clusters (Supplementary Fig. S2F), indicating that EMT is a necessary step for cerebellum metastasis. HER2 is reported to induce TGFβ/SMAD signaling to further activate Snail and Snail2 (47), and this mechanism aligns with the Erbb2–Tgfb1–Snail/Snail2/Twist1 cascade we observed.

In summary, inflammation in the TME of HER2^+^ BCBM has a potential role in tumor development. Understanding the complex interactions between HER2 signaling and the immune system is crucial for developing effective immunotherapeutic strategies and improving outcomes for patients with HER2^+^ BCBMs.

### IL34 secretion by breast cancer cells induces ARG1^+^ macrophages, and a blocking antibody reduces BCBM growth

We hypothesized that cytokines or chemokines specifically secreted by the tumor organoid cells could induce macrophage polarization at the invading edge in the BCBM models. The spatial transcriptomics data showed that *IL34* was highly expressed in the tumor area (Fig. 6A). Cerebellar neurons do not express IL34, and *IL34* knockout mice do not show changes in the microglia cell number in the cerebellum and brainstem (48). Therefore, we tested whether the breast cancer cells expressed IL34 protein using an ELISA assay. IL34 was detected in protein lysates from HP breast cancer cells but not from C57BL/6 mouse brain astrocytes or microglia (Fig. 6B). BMDMs from C57BL/6 mice were incubated with conditioned media from the HP breast cancer cells, and Arg1 protein expression was induced (Fig. 6C).

Cerebellar microglia homeostasis requires CSF1R, but they are not IL34-dependent (49). Microglia in the mouse cerebellum show distinct transcriptional profiles compared with other cell types (50). To assess how macrophages and microglia are spatially and functionally distributed in HER2^+^ BCBM, we examined their localization across tumor and invasion clusters (Supplementary Fig. S3A; Figs. 2C and 5E). ARG1, a marker of immunosuppressive macrophages (41), was enriched at the invasion border near normal cerebellum, whereas other macrophage/microglia markers (*CD11b*, *Iba1*, *Tmem119*, *Cd68*, and *Cd45*) were primarily found in tumor clusters (Supplementary Fig. S3A; Fig. 4D). *Camk2a*, a microglial functional marker, was restricted to normal brain regions (Supplementary Fig. S2A). Chemokines like *IL4*, *CCL2*, and *CCR2* were upregulated in tumor and invasion zones (Supplementary Fig. S3B), suggesting a role in macrophage recruitment and polarization. *IL34* expression correlated with ARG1^+^ macrophages at the invasion edge.

We confirmed IL34 and ARG1 expression at invasion borders in BCBM, whereas only sparse expressions were seen in primary HP breast tumors and metastatic lung lesions (Supplementary Fig. S4A and S4B). Conditioned media from HP tumor organoids induced macrophage-like morphology in BMDMs (51) and BV2 microglia (Supplementary Fig. S4C). After 24-hour coculture, both cell types showed increased ARG1 (Fig. 6C; Supplementary Fig. S4D) and IL34 expression (Supplementary Fig. S4E), confirming the ability of tumor-derived signals to drive immunosuppressive macrophage polarization. Additionally, we treated BV2 cells with recombinant murine IL34 and observed increased ARG1 expression (Supplementary Fig. S5).

CSF1R is the receptor for IL34, and a blocking antibody to mouse CSF1R is available (52, 53). We have already demonstrated that monoclonal antibodies can penetrate *in vivo* into these cerebellar BCBMs (Fig. 3). To model the treatment of brain metastases, we treated mice with established cerebellar BCBM with this CSF1R blocking antibody, and we observed BCBM shrinkage in three of four (75%) mice (Fig. 6D and E; Supplementary Fig. S6A and S6B). This was a short-term experiment with a 12-day duration of antibody treatment. In contrast, control mice showed dramatic tumor growth over the same time period (Fig. 6D and E; Supplementary Fig. S6A and S6B). MRI images of representative mice show BCBM shrinkage with CSF1R antibody treatment and BCBM growth in the control group (Fig. 6E). Furthermore, we performed CRISPR-mediated knockout in HP breast cancer cells with three independent guide RNAs. Two of the guide RNAs (KO #1 and 3) produced complete abrogation of IL34 production, as judged by IL34 ELISA (Supplementary Fig. S6C). We then injected these IL34 knockout breast cancer organoids into the cerebellum and observed dramatically reduced tumor growth compared with parental HP breast cancer organoids (Fig. 6F). These results suggest that IL34 production by breast cancer cells is required for BCBM growth. Furthermore, blocking IL34-CSF1R signaling between breast cancer cells and macrophages is a potential novel immunotherapy to treat brain metastasis and could be combined with existing treatments for HER2^+^ brain metastases.

### IL34 expression in human BCBM

**Figure 7.**
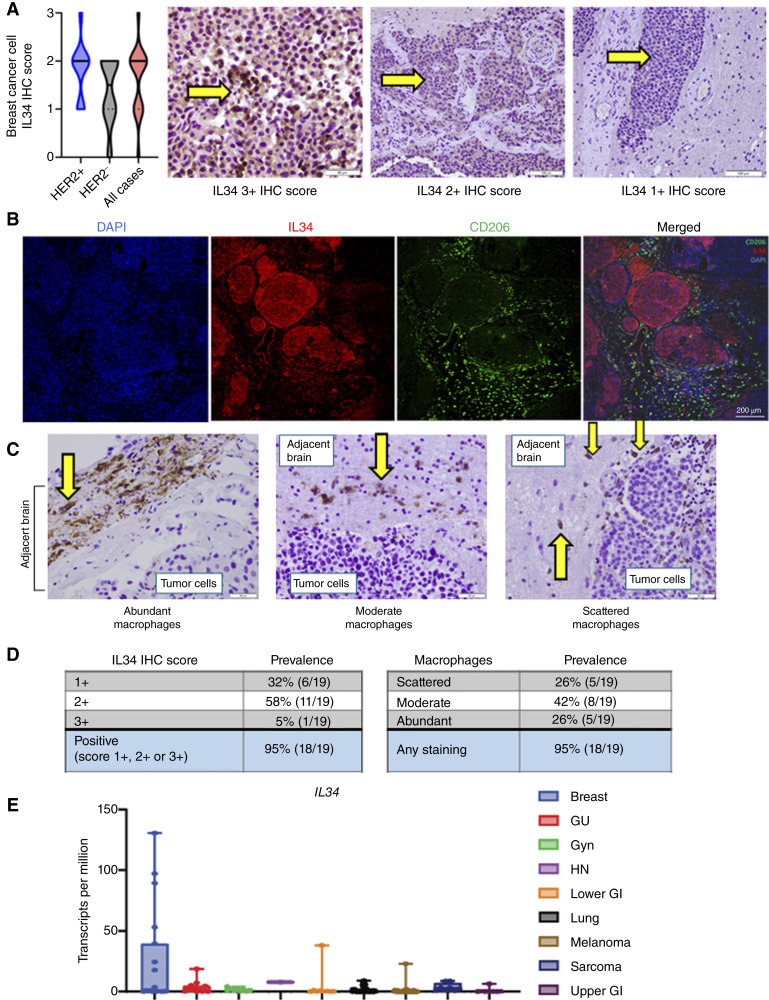
IL34 expression and CD163^+^ macrophages in human BCBM samples. **A,** IHC staining of human IL34 on human BCBM samples (*N* = 19). **B,** IF costaining of CD206 and IL34 on human BCBM samples (*N* = 8). **C,** CD163 IHC on human BCBM samples (*N* = 19). **D,** Summary of the IHC results (*N* = 19). **E,***IL34* mRNA expression level among the University of Michigan PDX samples (*N* = 137). GI, gastrointestinal; GU, genitourinary; Gyn, gynecological; HN, head and neck cancers.

The effectiveness of knocking out *IL34* in mice prompted us to determine whether IL34 is expressed in human BCBMs. We obtained two independent human sample datasets to answer this question. The first dataset consists of human neurosurgical resection samples obtained from the WUSM CNS tumor bank. We obtained FFPE blocks from 19 patients who underwent resection of brain metastasis from breast cancer, and we stained them for IL34 and the human macrophage markers CD163 and CD206. Both human CD163 and CD206 are markers of an immunosuppressive, polarized macrophage population corresponding to murine Arg1^+^ macrophages. Of these 19 BCBM cases, 11 were HER2-positive and 8 were HER2-negative. Of the HER2-negative cases, the majority were ER^+^ either in the primary tumor or in brain metastasis. We found strong IL34 expression via IHC in the HER2-positive cases and also in many of the HER2-negative cases (Fig. 7). Strong IL34 expression was seen regardless of which region of the brain the metastasis came from. Across all sites and all 19 samples, 58% (11/19) showed an IL34 IHC score of 2+, and any IL34 staining was seen in 95% of the cases (Fig. 7D). The case with the strongest IL34 staining (3+ IHC score) was a HER2-positive case resected from the parietal lobe of a patient (Fig. 7A and D).

Corresponding to the prior animal results, we also found macrophages in close proximity to the IL34^+^ breast cancer cells (CD206 staining in Fig. 7B and CD163 staining in Fig. 7C). These human macrophages are likely immunosuppressive macrophages that favor tumor progression, angiogenesis, and metastasis (54). Abundant CD163^+^ macrophages were seen in approximately one quarter of patient cases (26%, 5/19), and moderate or scattered macrophages at the tumor–brain interface were seen in 42% and 26% of cases, respectively (Fig. 7C and D). The presence of these macrophages was observed regardless of the HER2 status or the region of the brain from which the metastasis was resected (Fig. 7C and D).

We sought to validate these findings in a second dataset, which consisted of a collection of approximately 137 PDXs of brain metastases made by researchers at the University of Michigan (55). These PDXs were generated from resected brain metastases from solid tumors. Of these 137 distinct PDX models, 15 PDXs were derived from patients with BCBMs. RNA-seq was performed on these PDXs, and strikingly, we found elevated *IL34* mRNA expression (>4.9 transcripts per million) in a subset of BCBM cases (Fig. 7E). Six of 15 (40%) BCBMs had elevated *IL34* expression, whereas only 22 of 137 (16%) of other solid tumor PDX models had elevated *IL34*, which is a statistically significant difference (*P* < 0.05, Fisher exact test). These high *IL34* mRNA expression cases were seen in both triple-negative breast cancer and HER2-positive breast cancer cases.

## Discussion

In this article, we generated a novel, immunocompetent animal model for BCBM by transplanting cancer organoids from one B6 mouse to another syngeneic B6 mouse. We identified an essential role for Arg1^+^ macrophages at the invading edge of the metastasis, and we demonstrated that inhibiting these macrophages with a CSF1R-blocking antibody or by knocking out IL34 production from the breast cancer cells impaired metastasis growth and produced tumor shrinkage. Although there have been considerable advances in the treatment of HER2^+^ BCBM, including the antibody–drug conjugate trastuzumab deruxtecan (29, 56), the tyrosine kinase inhibitor tucatinib (57), and focused radiotherapy techniques (58), most patients with HER2^+^ BCBM will die a neurologic death from progressive brain metastasis (56). Therefore, new strategies and approaches to treat brain metastases are needed. Our results with targeting IL34–CSF1R signaling potentially provide such a new approach.

Inflammation is one of the hallmarks of cancer (59), and numerous studies have examined the role of tumor-associated macrophages (60). Attempts to inhibit or deplete tumor-associated macrophages with CSF1R tyrosine kinase inhibitors such as BLZ945 or JNJ-40346527 have unfortunately been unsuccessful (61, 62), possibly because of the activation of parallel or redundant pathways or off-target effects on closely related tyrosine kinases (63). Targeting IL34 or CSF1R with a monoclonal antibody has a number of potential advantages. First, antibody drugs often have greater affinity and specificity for the target protein (64). Second, monoclonal antibodies typically have a much longer half-life than small-molecule kinase inhibitors, thereby providing sustained target inhibition (65). Third, immunologic effects of antibody binding, such as antibody-dependent cellular cytotoxicity and antibody-dependent cellular phagocytosis, provide additional mechanisms for drug action beyond the kinase activity inhibition produced by small-molecule kinase inhibitors (64). The recent FDA approval of axatilimab for the treatment of another indication, chronic graft-versus-host disease (13), offers a new class of targeted therapies for CSF1R and tumor-associated macrophages.

These data suggest that IL34 production is a widespread property of BCBM. We observed IL34 production across all three breast cancer subtypes (HER2-positive, hormone receptor positive–HER2 negative, and triple-negative breast cancer), and we observed it in metastasis to different brain regions. Although we started this project to better understand HER2^+^ BCBM to the cerebellum, the broad finding of IL34 production by many BCBMs provides a new therapeutic target. Targeting inflammatory cytokines with antibodies and other novel therapeutics may improve the treatment of patients with brain metastasis.

### Limitations

We acknowledge that IL34 could act by additional mechanisms beyond polarizing and recruiting macrophages. IL34 can promote angiogenesis and act in an autocrine mechanism to stimulate the migration and invasion of cancer cells (66, 67). Whether to target IL34 or its receptor CSF1R to treat BCBM is an open question. The compensatory mechanisms to CSF1R inhibition have been identified (61), and therefore, targeting IL34 directly could be a better option for BCBM treatment.

We further acknowledge that direct injection of breast cancer cells into the cerebellum bypasses the intravasation and extravasation steps in the metastatic cascade. However, direct injection is the most experimentally tractable method to study cerebellar metastasis, and it provides highly useful information about the changing microenvironments around a growing metastasis. We also acknowledge that this study does not compare cerebellum metastasis with other brain regions. We plan to perform this comparison in our next study. Finally, spatial transcriptomics platforms and methods are rapidly evolving. In late 2024, 10x Genomics released the Visium HD platform, which provides up to 2-μm resolution (68, 69). The availability of single-cell or near-single-cell spatial techniques will provide further understanding of brain metastases.

In conclusion, this study provides valuable information about how inflammation affects cancer invasion in BCBM and demonstrates that IL34 production by the breast cancer cells recruits or polarizes Arg1^+^ macrophages at the invading edge of BCBM and is required for BCBM growth.

## Supplementary Material

Figure S1 Characterization of murine breast cancer organoids.

Figure S2 Spatial gene expression in mouse BCBM.

Figure S3 Spatial feature plot of macrophage markers and related chemokines and cytokines.

Figure S4 IL34-induced ARG1 expression in macrophages at the invading edge of BCBM.

Figure S5 IL34 induced ARG1 expression in BV2 cells.

Figure S6 Effect of CSF1R blocking antibody and IL34 CRISPR knockout.

Table S1 Average_Expression in clusters

Table S2 Differentially Expressed Genes

Table S3 Pathway Genes

## Data Availability

Spatial transcriptomics data generated in this study are deposited in the Gene Expression Omnibus, accession code GSE325996. scRNA-seq data on the HP organoids are deposited in the Gene Expression Omnibus, accession code GSE326070. Open source code used is available at Code Ocean (https://codeocean.com/capsule/7986416/tree/v1).
