# Supplementary material for: Interleukin-34–Induced Arg1+ Macrophages Play a Key Role in Breast Cancer Brain Metastasis
Source: Cancer Res Commun. 2026 Jun 12;6(6):1388–404. doi: 10.1158/2767-9764.CRC-25-0639 (PMC13261624; doi:10.1158/2767-9764.CRC-25-0639)
Supplement: Figure S1 — Characterization of murine breast cancer organoids. [file crc-25-0639_figure_s1_suppsf1.pdf]

Figure S1

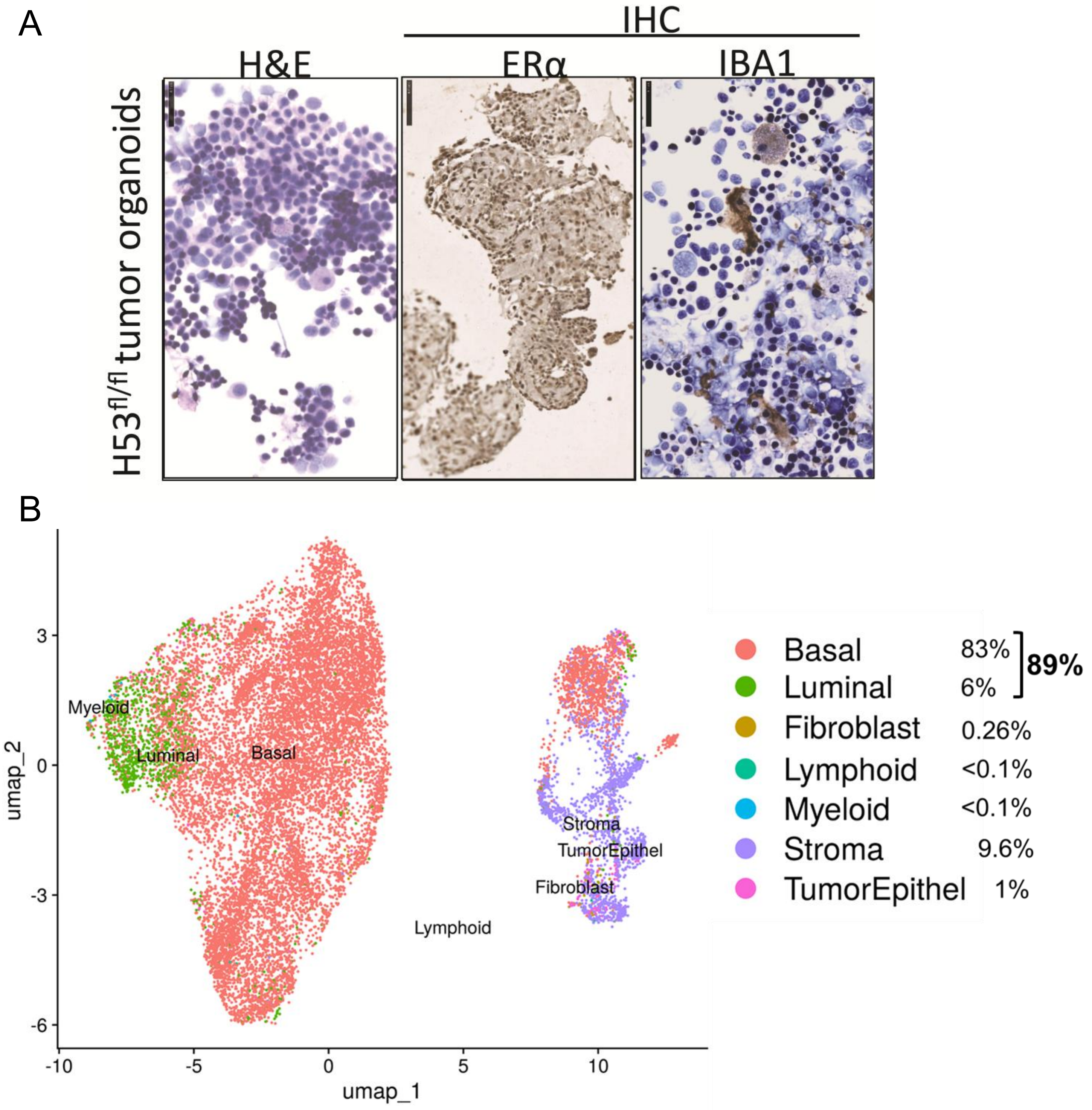

Figure S1. Characterization of murine breast cancer organoids.
A, H&E, and IHC stained with ER***α***, IBA1 antibodies in H53fl/fl tumor organoid cells. The scale bars in high-power images are 500 nm. **B,** UMAP of single-cell RNA sequencing result shows cell type clusters and percentage of the corresponding cell types.
