# Supplementary material for: Interleukin-34–Induced Arg1+ Macrophages Play a Key Role in Breast Cancer Brain Metastasis
Source: Cancer Res Commun. 2026 Jun 12;6(6):1388–404. doi: 10.1158/2767-9764.CRC-25-0639 (PMC13261624; doi:10.1158/2767-9764.CRC-25-0639)
Supplement: Figure S2 — Spatial gene expression in mouse BCBM. [file crc-25-0639_figure_s2_suppsf2.pdf]

Figure S2

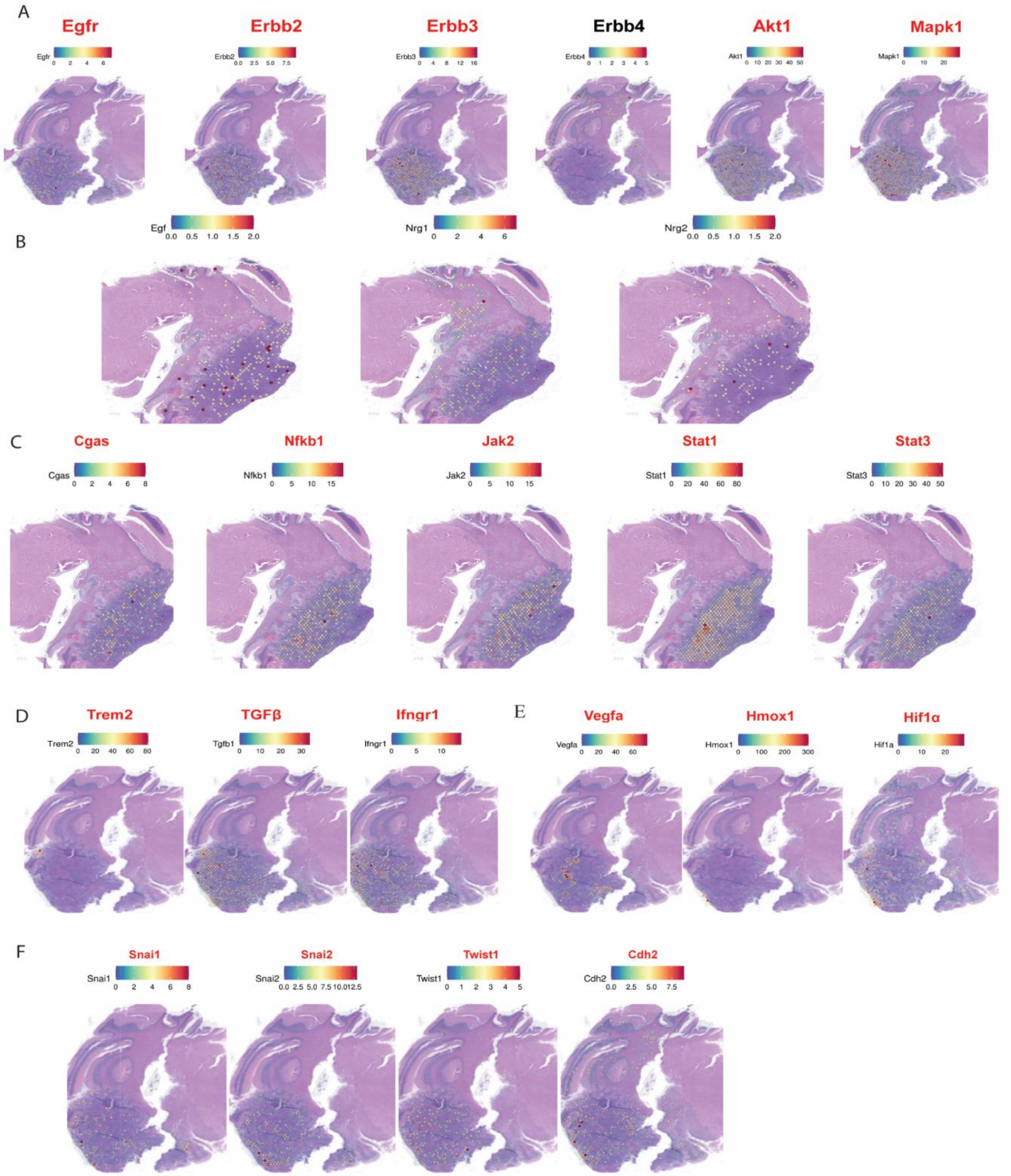

Fig S2. Spatial gene expression in mouse BCBM.

A, Spatial feature plot of key genes and factors related to HER2 signaling in the representative tissue sample. **B,** Spatial feature plot of HER family ligands in the representative tissue sample. **C,** Spatial feature plot of key genes or transcription factors regulating inflammation signaling in the representative tissue sample. **D,** Spatial feature plot of macrophage activators in the representative tissue sample. **E,** Spatial feature plot of key genes or transcription factors regulating angiogenesis in the representative tissue sample. **F,** Spatial feature plot of key genes or transcription factors regulating EMT (epithelial-mesenchymal transition) signaling in the representative tissue sample.
