# Supplementary material for: Interleukin-34–Induced Arg1+ Macrophages Play a Key Role in Breast Cancer Brain Metastasis
Source: Cancer Res Commun. 2026 Jun 12;6(6):1388–404. doi: 10.1158/2767-9764.CRC-25-0639 (PMC13261624; doi:10.1158/2767-9764.CRC-25-0639)
Supplement: Figure S3 — Spatial feature plot of macrophage markers and related chemokines and cytokines. [file crc-25-0639_figure_s3_suppsf3.pdf]

Figure S3

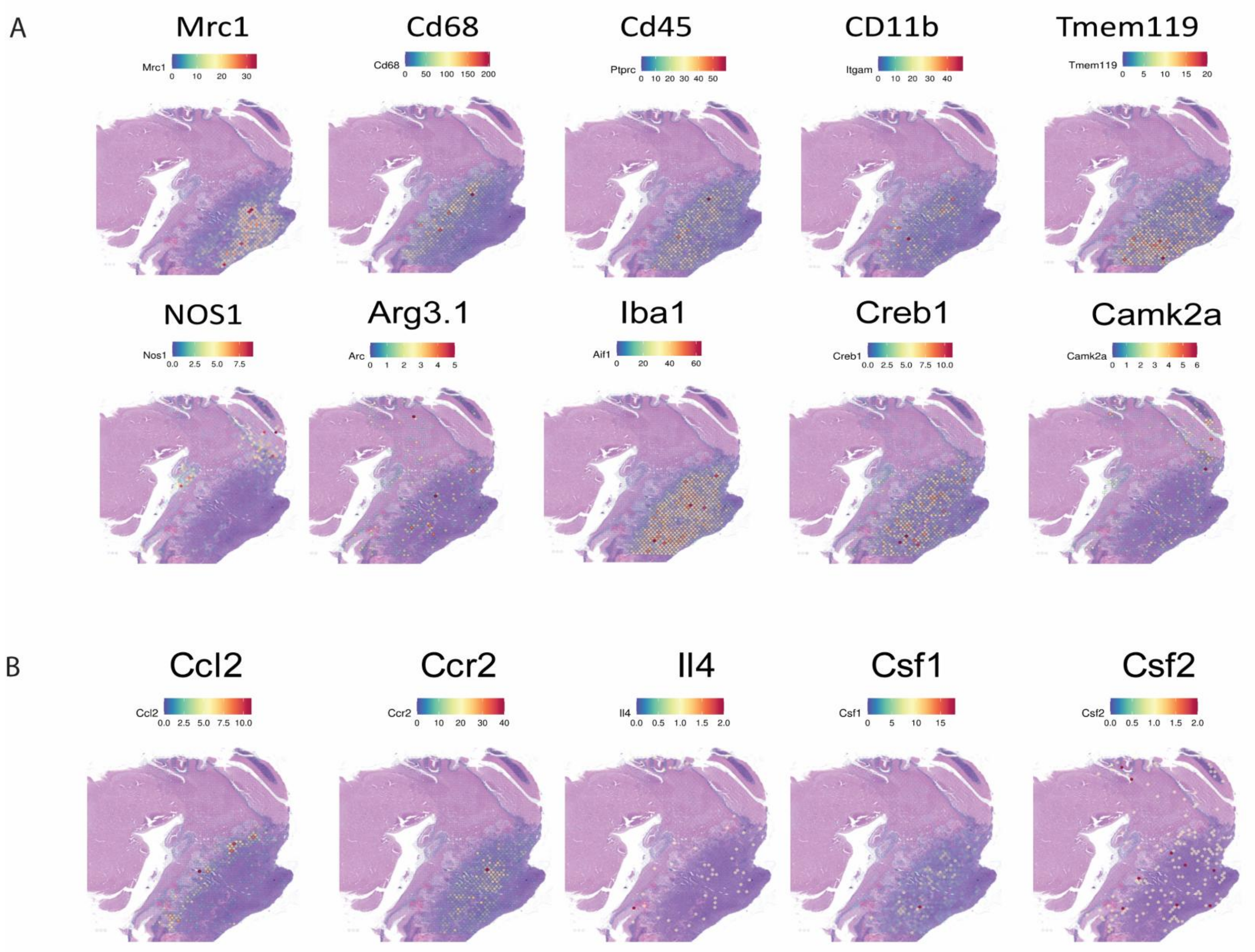

Figure S3. Spatial feature plot of macrophage markers and related chemokines and cytokines.

A, Spatial feature plot of markers for macrophage and microglial cells in the representative tissue sample. **B,** Spatial feature plot of key chemokines and cytokines regulates macrophages in the representative tissue sample.
