# Supplementary material for: Interleukin-34–Induced Arg1+ Macrophages Play a Key Role in Breast Cancer Brain Metastasis
Source: Cancer Res Commun. 2026 Jun 12;6(6):1388–404. doi: 10.1158/2767-9764.CRC-25-0639 (PMC13261624; doi:10.1158/2767-9764.CRC-25-0639)
Supplement: Figure S4 — IL34-induced ARG1 expression in macrophages at the invading edge of BCBM. [file crc-25-0639_figure_s4_suppsf4.pdf]

Figure S4

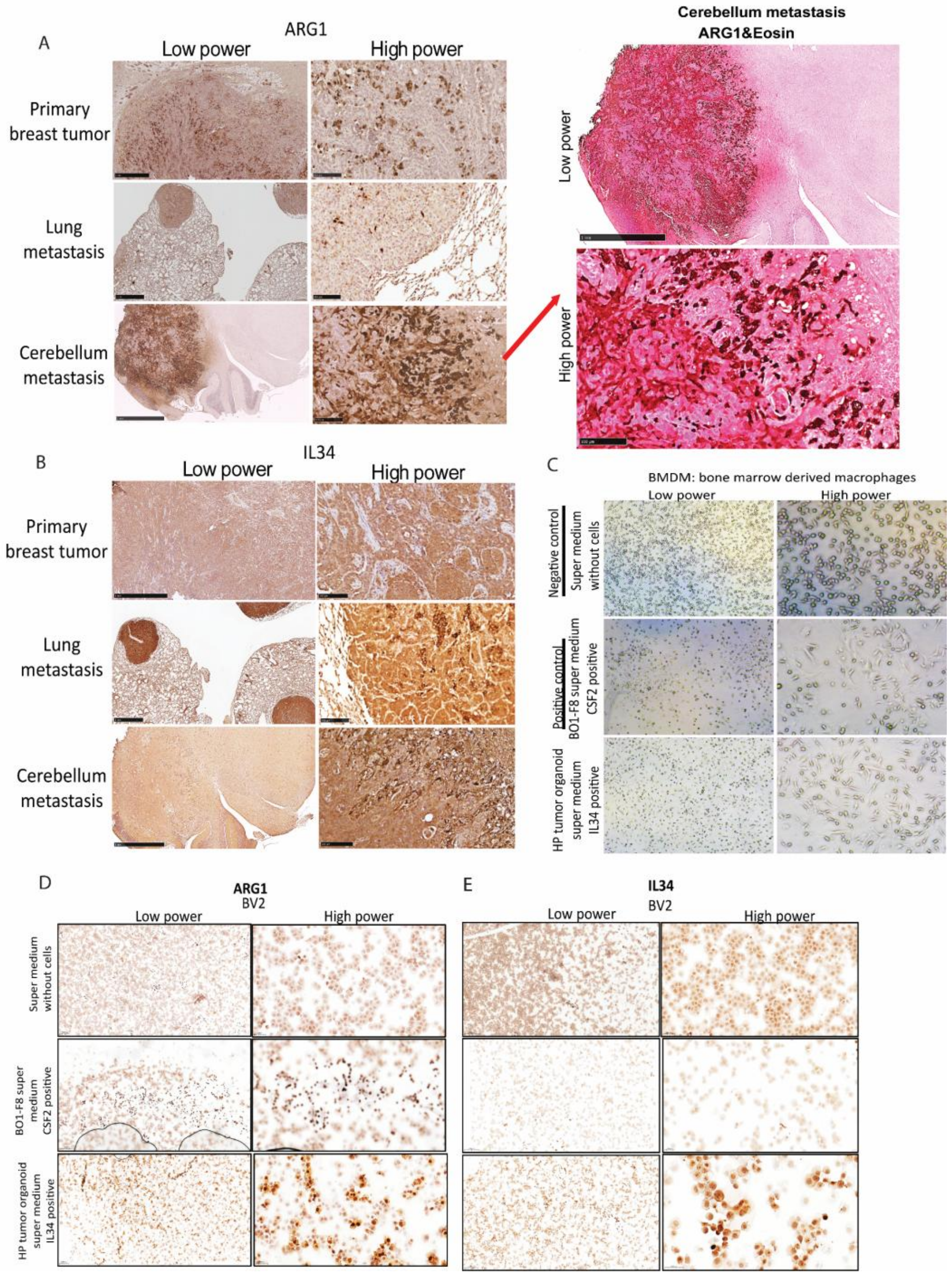

Fig S4. IL34-induced ARG1 expression in macrophages at the invading edge. **A,** Left: IHC staining of ARG1 in primary breast tumors, lung metastatic tumors, and cerebellum metastatic tumors from tissue samples. Right: IHC staining of ARG1 and eosin staining in cerebellum metastatic tumors from the tissue sample. **B,** IHC staining of IL34 in primary breast tumors, lung metastatic tumors, and cerebellum metastatic tumors from tissue samples. **C,** The mouse BMDM was cultured with conditioned media supernatant from BO1(51) cells or HP tumor organoid cells for 24 hours, and the bright field image shows the cell morphology change. **D,** The mouse BV2(69) was cultured with conditioned media supernatant from BO1(51) cells or HP tumor organoid cells for 24 hours, and the IHC staining of ARG1 was performed on the cytospin slides of the co-cultured cells. **E,** The mouse BV2 was cultured with a conditioned media supernatant from BO1(51) cells or HP tumor organoid cells for 24 hours, and the IHC staining of IL34 was performed on the cytospin slides of the co-cultured cells.
