# Supplementary material for: Interleukin-34–Induced Arg1+ Macrophages Play a Key Role in Breast Cancer Brain Metastasis
Source: Cancer Res Commun. 2026 Jun 12;6(6):1388–404. doi: 10.1158/2767-9764.CRC-25-0639 (PMC13261624; doi:10.1158/2767-9764.CRC-25-0639)
Supplement: Figure S5 — IL34 induced ARG1 expression in BV2 cells. [file crc-25-0639_figure_s5_suppsf5.pdf]

Figure S5

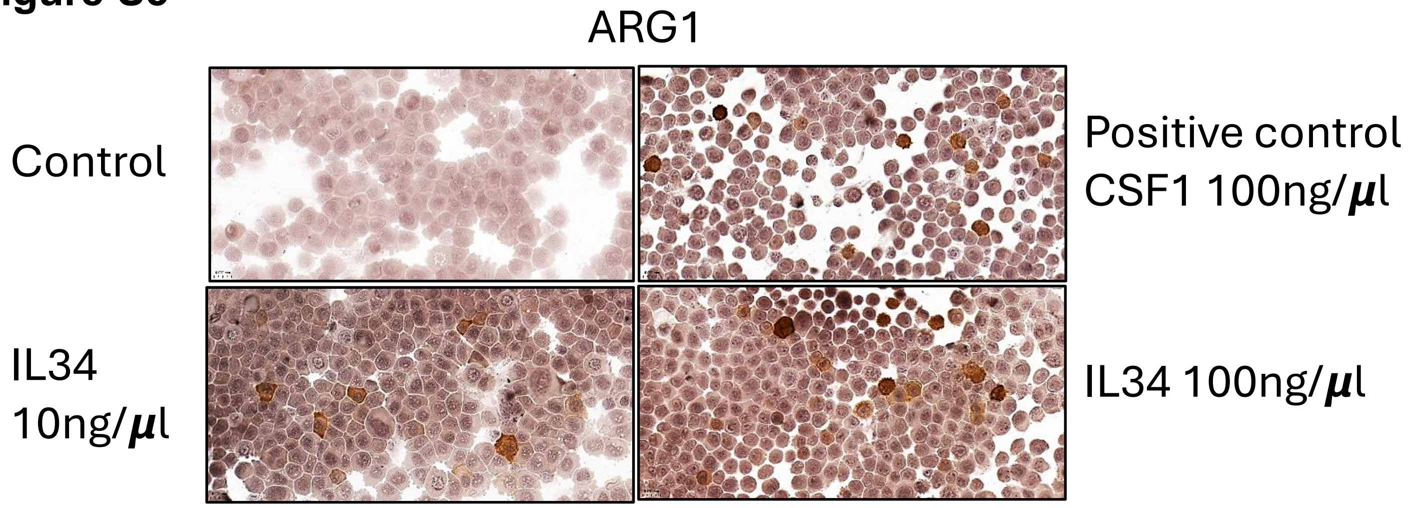

Figure S5. IL34 induced ARG1 expression in BV2 cells.
Mouse BV2 cells were treated with recombinant CSF1 or IL34 for 24 hours, and IHC staining of ARG1 was performed as per Fig S4D.
