# Supplementary material for: Interleukin-34–Induced Arg1+ Macrophages Play a Key Role in Breast Cancer Brain Metastasis
Source: Cancer Res Commun. 2026 Jun 12;6(6):1388–404. doi: 10.1158/2767-9764.CRC-25-0639 (PMC13261624; doi:10.1158/2767-9764.CRC-25-0639)
Supplement: Figure S6 — Effect of CSF1R blocking antibody and IL34 CRISPR knockout. [file crc-25-0639_figure_s6_suppsf6.pdf]

Figure S6

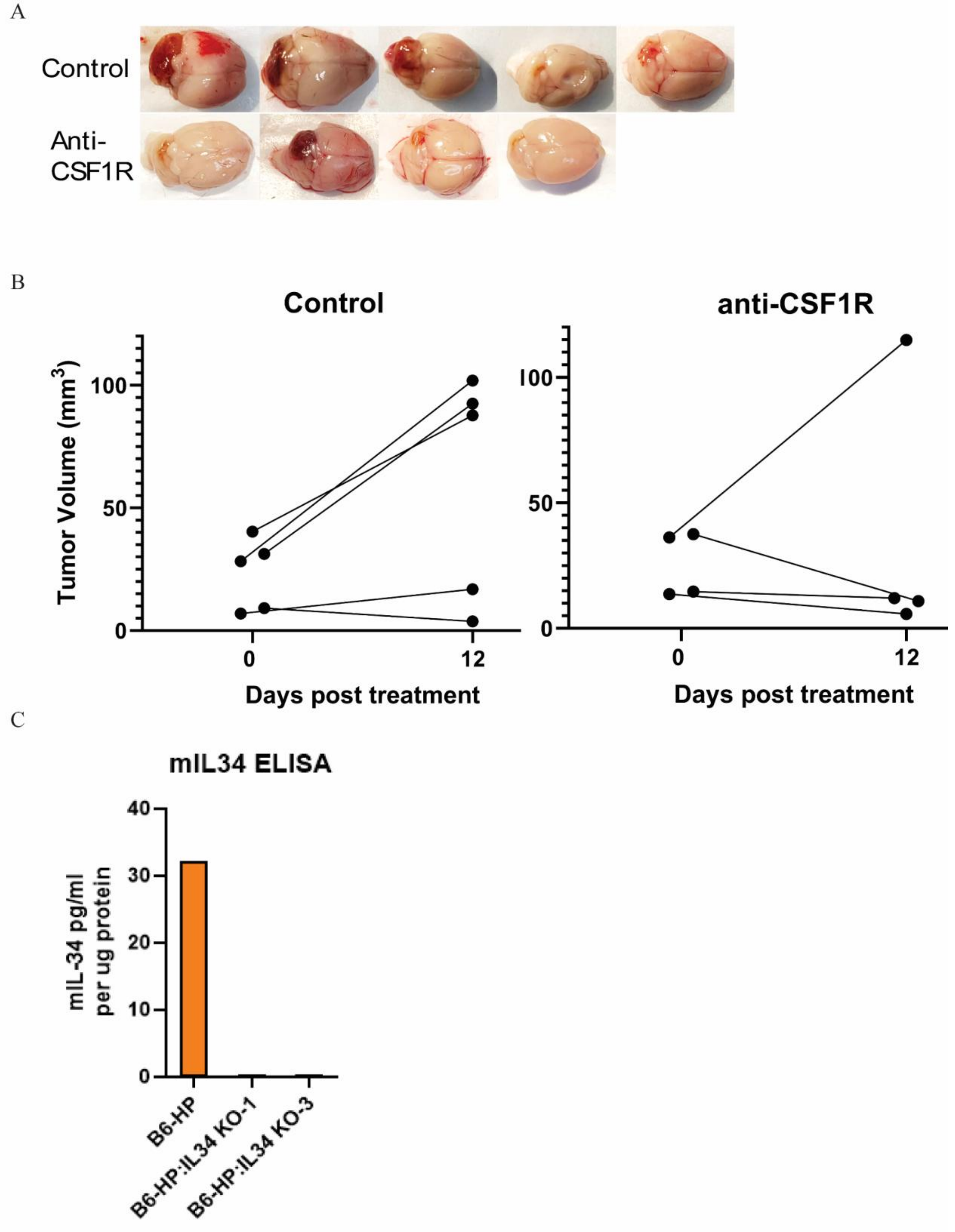

Figure S6. Effect of CSF1R blocking antibody and *IL34* CRISPR knockout.
A, Brightfield images of dissected cerebellum tumor from control and anti-CSF1R mAb groups. **B,** tumor volume change in the control and anti-CSF1R mAb groups measured by MRI at day 0 and day 12 post-treatment. **C,** *IL-34* production was measured in tumor organoids lines using a mouse *IL-34* ELISA.
